# Comparison of Measurements with Finite-Element Analysis of Silicon-Diaphragm-Based Fiber-Optic Fabry–Perot Temperature Sensors

**DOI:** 10.3390/s19214780

**Published:** 2019-11-03

**Authors:** Rongkun Wang, Xuejian Xie, Xiangang Xu, Xiufang Chen, Longfei Xiao

**Affiliations:** State Key Laboratory of Crystal Materials, Shandong University, Jinan 250100, China; 201511917@mail.sdu.edu.cn (R.W.); xiexj@sdu.edu.cn (X.X.); xxu@sdu.edu.cn (X.X.)

**Keywords:** fiber-optic Fabry–Perot sensor, finite-element analysis, temperature sensor

## Abstract

Silicon-diaphragm-based fiber-optic Fabry–Perot sensors with different intracavity pressures were fabricated by anodic bonding and microelectromechanical techniques. The thermal stress and thermal expansion of the Fabry–Perot (FP) sensor caused by high-temperature bonding and temperature change were simulated by finite-element analysis. The calculated thermal stress is largest in the center and edge regions of the resonance cavity, reaching from 2 to 6 MPa. The reflection spectra and temperature sensitivity of the sensors were simulated by using a two-dimensional wave-optic model in Comsol. Theoretical calculations were also made for the FP cavity without considering silicon-diaphragm deformation and thermal stress. Four sensors with intracavity pressures of 0.01, 0.03, 0.04, and 0.05 MPa were tested at low temperatures, showing a high degree of consistency with the simulation results rather than theoretical calculation, especially for high intracavity pressure. This method is expected to aid the analysis of thermal stress generated during the bonding process and to facilitate better design and control of the temperature sensitivity of the sensor.

## 1. Introduction

In recent years, fiber-optic temperature and pressure sensors have attracted significant interest because of their unique advantages over conventional sensors, notably their compact size, high sensitivity, fast response, good stability, and immunity to electromagnetic interference [1,2,3,4,5,6,7,8,9,10,11]. In particular, fiber-optic Fabry–Perot (FP) interferometric sensors based on a pressure-sensitive diaphragm have been widely used for temperature measurements in industry, medical and power-electronics applications [12,13,14,15]. The material and geometry of the diaphragm are key factors determining the temperature sensitivity and monitoring range of these sensors. In the hope of producing more sensitive sensors, various materials have been used to fabricate fiber-optic FP sensors. The main considerations in choosing the diaphragm material of the sensor are the physical parameters of the material and the difficulty of diaphragm processing, including thinning and bonding. Silicon (or silica) is among the earliest and most widely used materials for fiber-optic FP sensors because of its excellent properties. These include good mechanical, chemical, and thermal stability [16,17,18,19], which gives the silicon-glass bonded sensor great advantages based on the high bonding strength and simple bonding process. With the discovery of new materials and the continuous improvement of processing technology, more materials are being tested in the fabrication of sensing diaphragms, such as silver [20,21], polymers, and two-dimensional materials [22,23,24], with the overall goal being higher sensitivity.

The resonance cavity of a silicon-diaphragm-based fiber-optic FP sensor is typically constructed by using a sealed air cavity formed by anodic bonding between silicon and Pyrex^®^ glass at high temperature. Due to the different thermal-expansion coefficients of silicon and Pyrex^®^ glass, thermal stress is generated in the silicon diaphragm from the bonding interface when the resonance cavity cools from bonding temperature to room temperature. The temperature sensitivity and linearity of the sensor is then affected by the thermal stress inside the silicon diaphragm and by the residual pressure in the sealed air cavity. 

In interference analysis inside the resonance cavity of fiber-optic FP sensors, the silicon diaphragm and the end face of the optical fiber served as reflecting mirrors. Multibeam interference fringe patterns occur when multiple beams with path differences impinge simultaneously on the resonance cavity. FP interference is explained by the theory of parallel-plate multibeam interference, which considers two strictly parallel mirrors and ignores absorption losses at the mirrors. However, a silicon diaphragm acting as one of the mirrors of a resonance cavity may be elastically deformed by a pressure difference. In addition, losses from the dispersion of light and material absorption are inevitable in real practice. Thus, multibeam interference analysis of FP sensors is extraordinarily complicated and hard to match with analytical solutions.

This paper reports on a finite-element analysis used to simulate the internal thermal stress and elastic deformation of a silicon diaphragm at different temperatures. Multibeam interference fringe patterns and reflected spectra from the optical-fiber FP sensor were calculated by using commercial analysis software. The relationship between temperature and wavelength shift was obtained by analyzing the position of the interference peak of the reflection spectrum at different temperatures. In addition, multibeam interference of fiber-optic FP sensors with different residual pressures in the resonance cavity was simulated to analyze the relationship between temperature sensitivity and residual pressure. To compare with the results of the finite-element simulation, we fabricated silicon-diaphragm-based optical-fiber FP sensors with similar diaphragm thicknesses with different residual pressures in the resonance cavity and subjected them to temperature tests.

## 2. Sensor Fabrication

**Figure 1:**
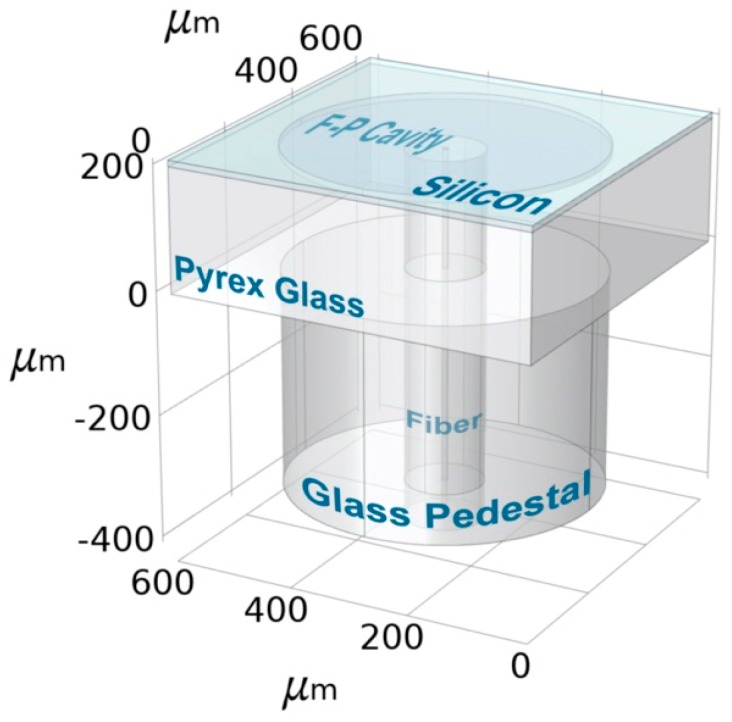
Schematic diagram of sensor diaphragm.

A silicon-diaphragm-based fiber-optic FP sensor was fabricated by surface and bulk microelectromechanical techniques, including chemical etching, anodic bonding, silicon-wafer thinning, and optical-fiber integration. Figure 1 shows the structure of the FP sensor. First, a patterned Ni-Au mask was prepared by electron-beam evaporation and photolithography on a Pyrex^®^ 7740# glass wafer with a thickness of 300 μm. A series of shallow cavities were etched on the glass surface by chemical etching with hydrogen fluoride. The radius and depth of the etching pit (i.e., the resonance cavity) were 250 and 4.5 μm, respectively. After removing the Ni-Au mask, a 4-inch <100>-oriented silicon wafer was used as a pressure-sensitive diaphragm for the sensor and was processed by anodic bonding with the Pyrex^®^ 7740# glass wafer at 450 °C. The initial thickness of the silicon wafer was 200 μm. After anodic bonding, the silicon wafer was thinned and polished by mechanical grinding and inductively coupled plasma etching to a thickness of 7 to 9 μm. Next, a single-mode fiber (Corning SMF28e) was inserted into the hole previously drilled at the center of the resonance cavity by femtosecond laser perforation. Finally, the fiber was attached to the sensor head and glass pedestal by using an ultraviolet adhesive applied in a glove box in which the pressure may be changed by adjusting the gas flow.

Upon anodic bonding of the silicon wafer to the Pyrex^®^ 7740# glass wafer, a large thermal stress is generated on the silicon diaphragm because of the mismatch in thermal expansion coefficients between the two materials. Their firm adhesion causes compressive or tensile stresses inside the silicon diaphragm during cooling. Therefore, the thermal response of the sensor is affected somewhat by thermal stress. Another consideration is how thermal expansion of the various parts of the sensor affects the elastic deformation of the silicon diaphragm, including the silicon material itself and any residual gas in the resonance cavity. 

## 3. Theoretical Analysis

**Figure 2:**
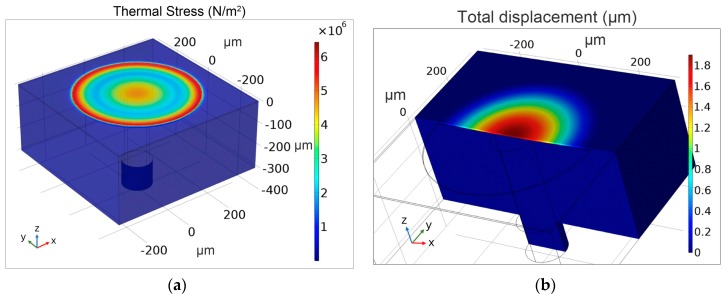
Simulation results for (**a**) thermal stress and (**b**) thermal expansion of the sensor head.

**Figure 3:**
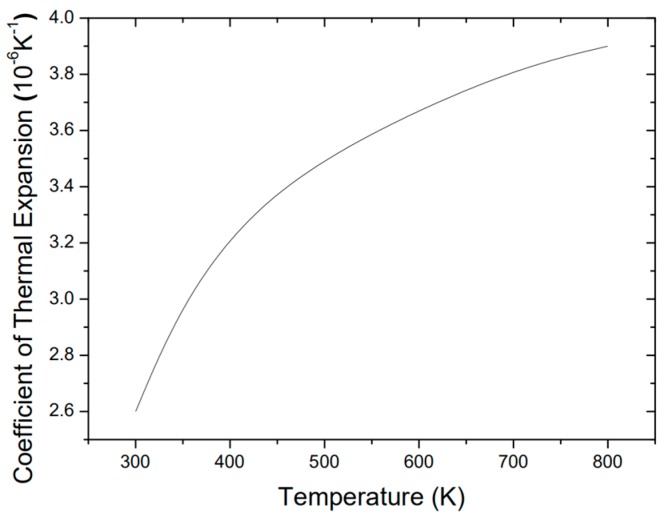
Thermal expansion coefficient of silicon.

In the finite-element analysis, the thermal stress and thermal expansion simulation of the sensing head was obtained by coupling the solid heat-transfer module to the solid mechanical module in Comsol. Figure 2a shows the simulated thermal stress in the sensor head after cooling from bonding temperature to room temperature. These results indicate that the largest thermal stress on the silicon diaphragm is in the center and near the edges of the FP cavity, where thermal stress varies from 2 to 6 MPa. Furthermore, the concave elastic deformation of the silicon diaphragm was calculated by using the thermal expansion of the sensor material and the gas in the resonance cavity (see Figure 2b). The coefficient of thermal expansion of the silicon used in the simulation is shown in Figure 3, and the thermal expansion coefficient of the Pyrex 7740# glass is determined to be a constant value that does not change with temperature [25].

**Figure 4:**
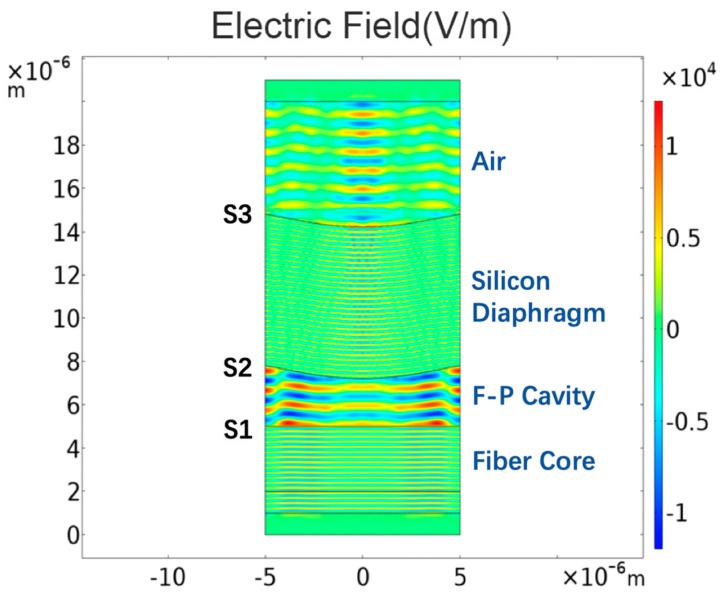
Simulation of distribution of electric-field intensity.

In this silicon-diaphragm-based FP sensor, the resonance cavity contains three reflecting mirrors: The inner and outer surfaces S2 and S3 of the silicon diaphragm and the end facet S1 of the optical fiber (see Figure 4). Three reflections are generated on each surface when light propagates through the optical fiber and F-P cavity. The traditional FP interference principle is based on the following assumptions: (1) The two reflecting mirrors as strictly parallel; (2) and any loss of light due to dispersion and absorption by the reflecting mirrors is ignored. However, in practice, deformation of the silicon diaphragm by pressure and absorption of light is inevitable. Thus, the finite-element analysis was used to simulate multibeam interference in the FP cavity of the sensor under different temperatures. 

In the wave-optics module, the entire analysis domain was set to the two-dimensional region through which light propagates. The refractive index and other physical parameters of the materials were introduced in each part of the model. The maximum and minimum grid sizes were 70 and 5 nm, respectively. The mode of incident light was set to plane wave incidence, and the left and right boundary conditions of the model were periodic boundary conditions. Note that the simulation used a two-dimensional model mainly to minimize the calculation and achieve a more precise scanning wavelength from which to analyze any shift in the interference peak; some error will inevitably enter the simulation of spectral reflectance. 

**Figure 5:**
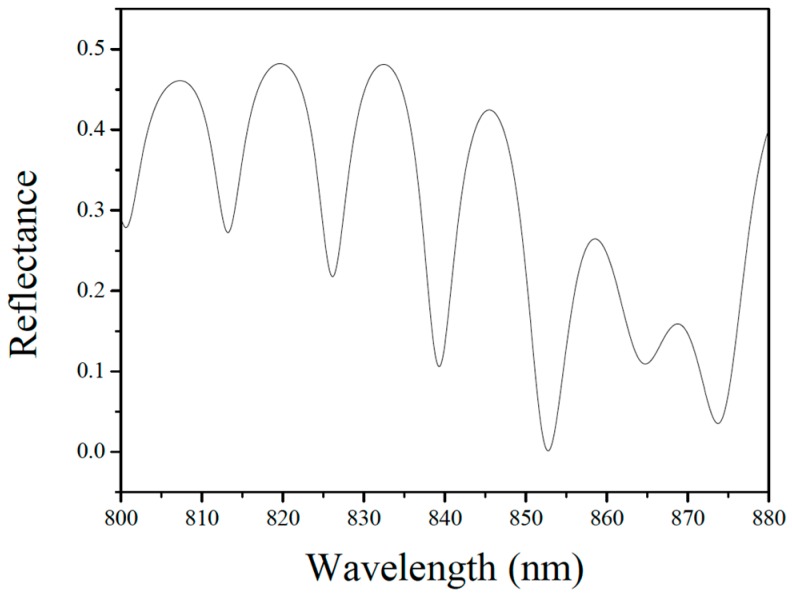
Simulated reflection spectrum of the sensor.

**Figure 6:**
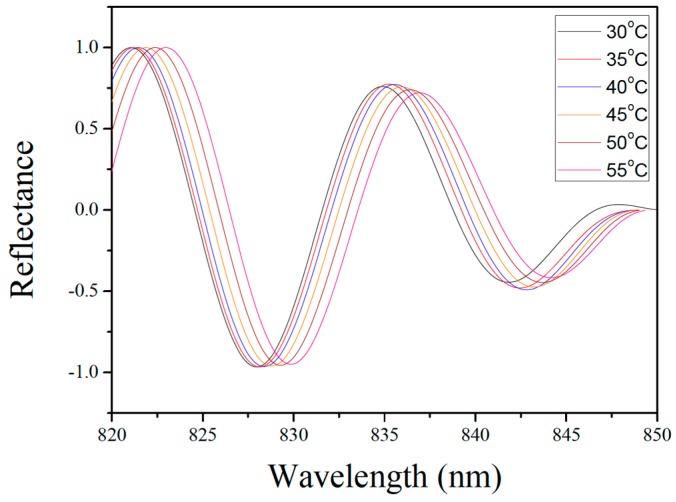
Simulated reflection spectra for different temperatures.

Figure 4 shows the electric field distribution in the fiber-optic FP sensor model at 850 nm. The model comprises a single-mode fiber core, the air inside the resonance cavity, the silicon diaphragm, and the exterior air. It should be noted that the deformation of the silicon diaphragm and the width of the model in Figure 4 were appropriately amplified to obtain a more intuitive electric field distribution result. In the actual simulation, the deformation of the silicon diaphragm was in the order of nanometers, and the width of the model was 8.2 μm. The model was subjected to parametric scanning covering different wavelengths to obtain the reflectance spectrum of the sensor (see Figure 5). Next, the elastic deformation of the silicon diaphragm in the thermal-expansion model was introduced into the wave-optics model to obtain the interference spectrum at different temperatures. The physical parameters in the model were 7 μm for the thickness of the silicon diaphragm; 4.5 and 250 μm for the depth and radius of the FP cavity, respectively; and 0.03 MPa for the initial gas pressure inside the FP cavity. The simulated reflection spectra in Figure 6 is the result of FFT and filtering for more intuitive analysis of wavelength shifts. 

**Figure 7:**
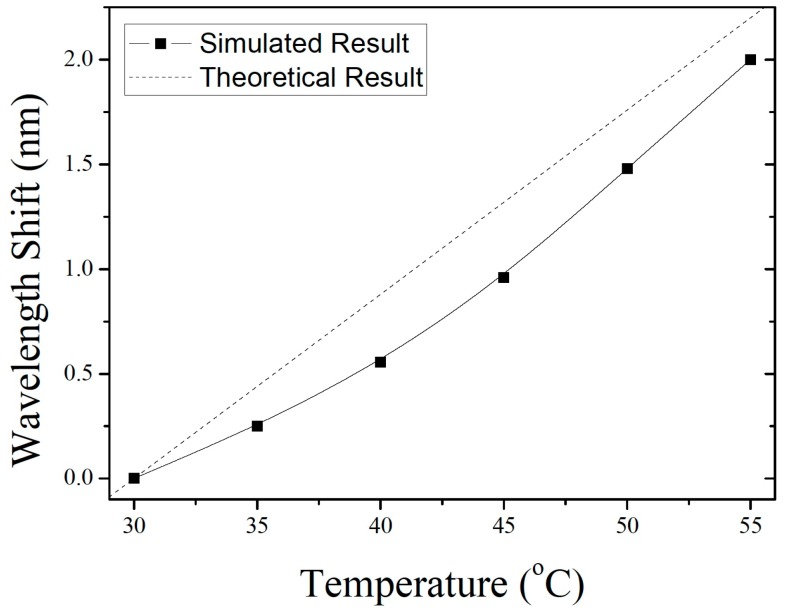
Simulation of temperature sensitivity of sensor: Wavelength shift versus temperature.

**Figure 8:**
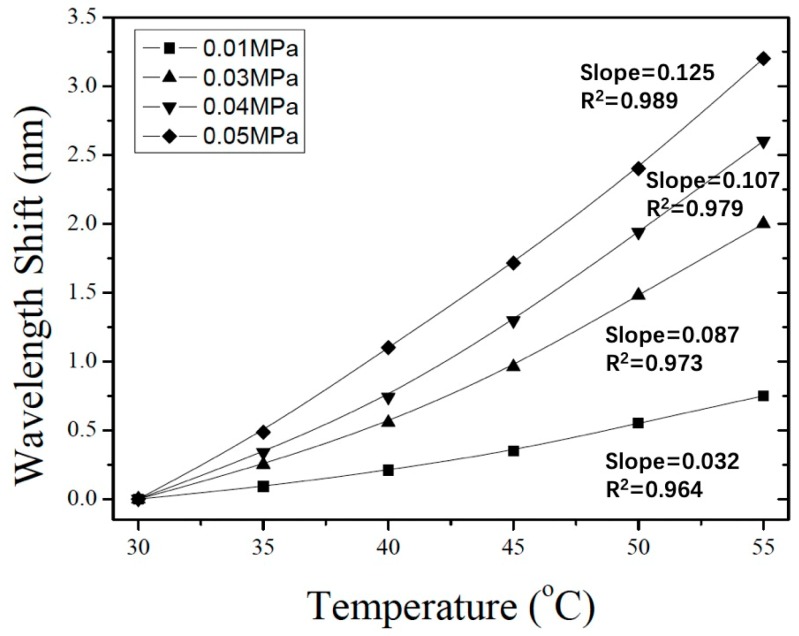
Simulation of temperature sensitivity of sensor for different intracavity pressures.

Figure 7 shows the wavelength shift as a function of temperature; these results give a temperature sensitivity of the FP sensor of 0.087 nm/°C. In addition, Figure 8 shows the results of temperature simulations with initial pressures of 0.01, 0.03, 0.04, and 0.05 MPa inside the FP cavity. The simulated temperature sensitivity of the different sensors are 0.032, 0.087, 0.107, and 0.125 nm/°C. Furthermore, the linearity of these sensors are 0.964, 0.973, 0.979, and 0.989, respectively. The simulation results show that, as the gas pressure in the FP cavity increases, both the temperature sensitivity and linearity of the sensors with the same silicon-diaphragm thickness also increase.

In the traditional theoretical model, the ideal FP interference formula is used without considering the elastic deformation of the silicon diaphragm caused by thermal stress under different temperatures. According to elastic mechanics, the deformation ω(r) of the silicon diaphragm a distance *r* from the center of the silicon diaphragm is

(1) $$\omega \left(r\right)=\frac{3\left(1-\nu^{2}\right){\left(a^{2}-r^{2}\right)}^{2}}{16Eh^{3}}\cdot P$$

where *a* and *h* the radius and thickness of the silicon diaphragm, respectively; ν and *E* are Poisson’s ratio and Young’s modulus for silicon, respectively; and *P* is the pressure on the silicon diaphragm.

According to Equation (1), the volume of the FP cavity is

(2) $$V=L_{0}\cdot \pi a^{2}-\int_{o}^{a}\omega \left(r\right)\cdot 2\pi r\cdot dr$$

where $L_{0}$ is the initial cavity length of the FP cavity. The relationship between cavity length and temperature is then obtained by combining the ideal-gas equation with Equations (1) and (2).

If the interference cavity of the sensor is considered to be an ideal FP cavity bounded by parallel mirrors and is analyzed by a dual-wave interference model, the wavelength shift can be written as Δλ=λ·ΔL/L, where ΔL is the variation in cavity length. Figure 7 shows the theoretical curve of wavelength versus temperature.

## 4. Experimental Setup

**Figure 9:**
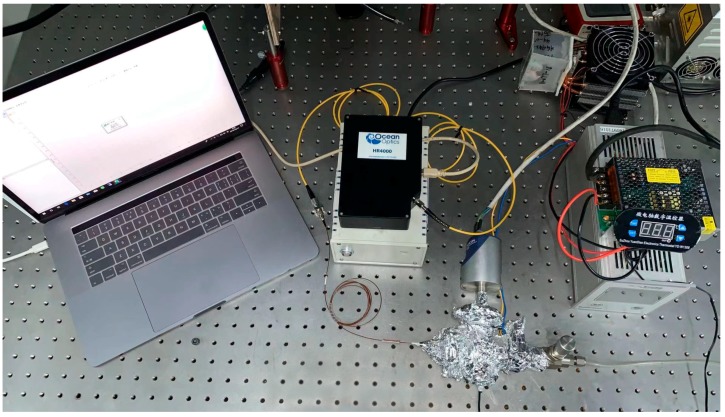
Experimental setup.

Figure 9 shows the experimental setup used for measuring the temperature response of the fabricated FP sensor. The spectrometer was an Ocean Optics HR4000 with an 800–878 nm grating and a 25 μm slit. The spectrometer was connected to a fiber-coupled LED (OPF372a) and the FP sensor by a circulator (Lfiber SR-0092). The fabricated sensor was sealed in an insulated chamber heated and monitored by a temperature controller. The spectrometer was connected to a computer for data acquisition and analysis.

## 5. Experimental Results

**Figure 10:**
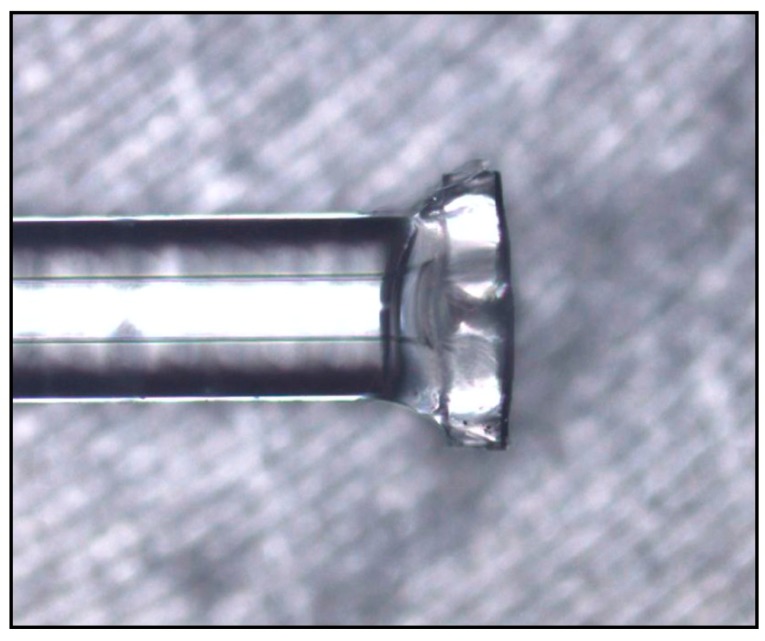
Optical-microscope image of sensor.

**Figure 11:**
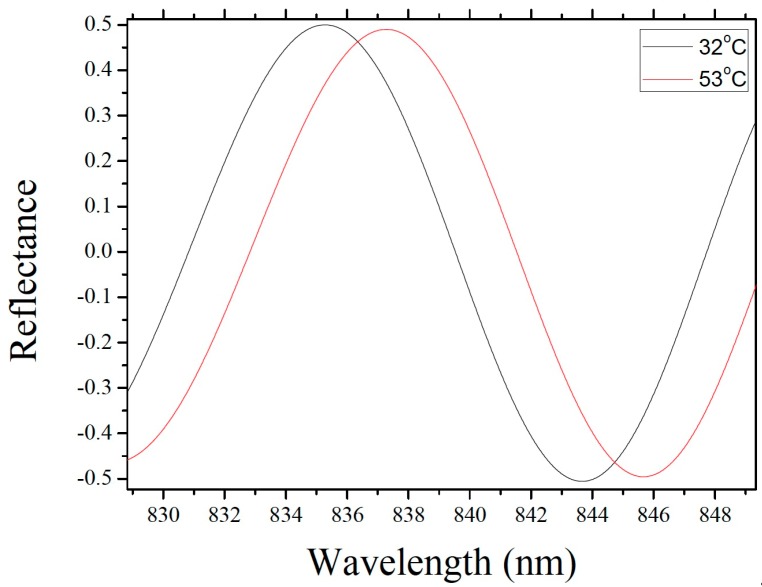
Reflection spectra at different temperatures.

**Figure 12:**
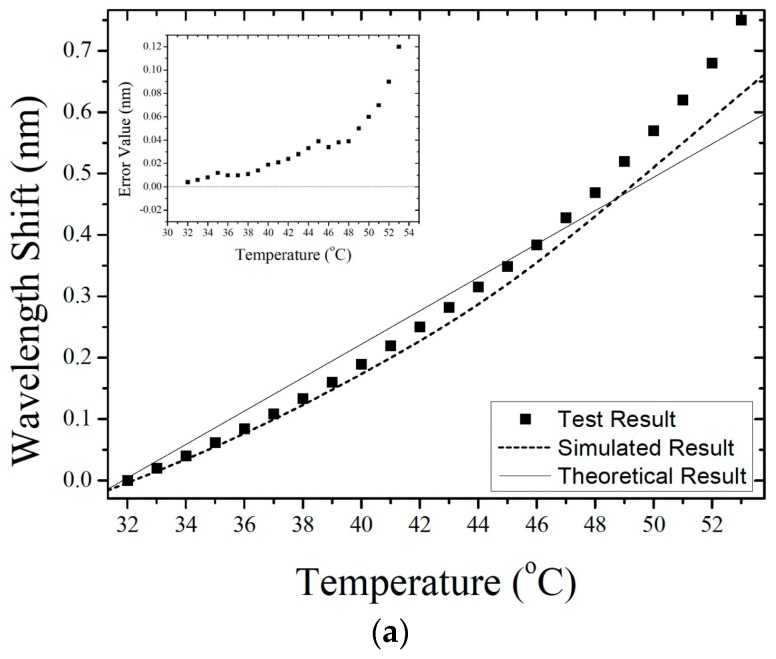

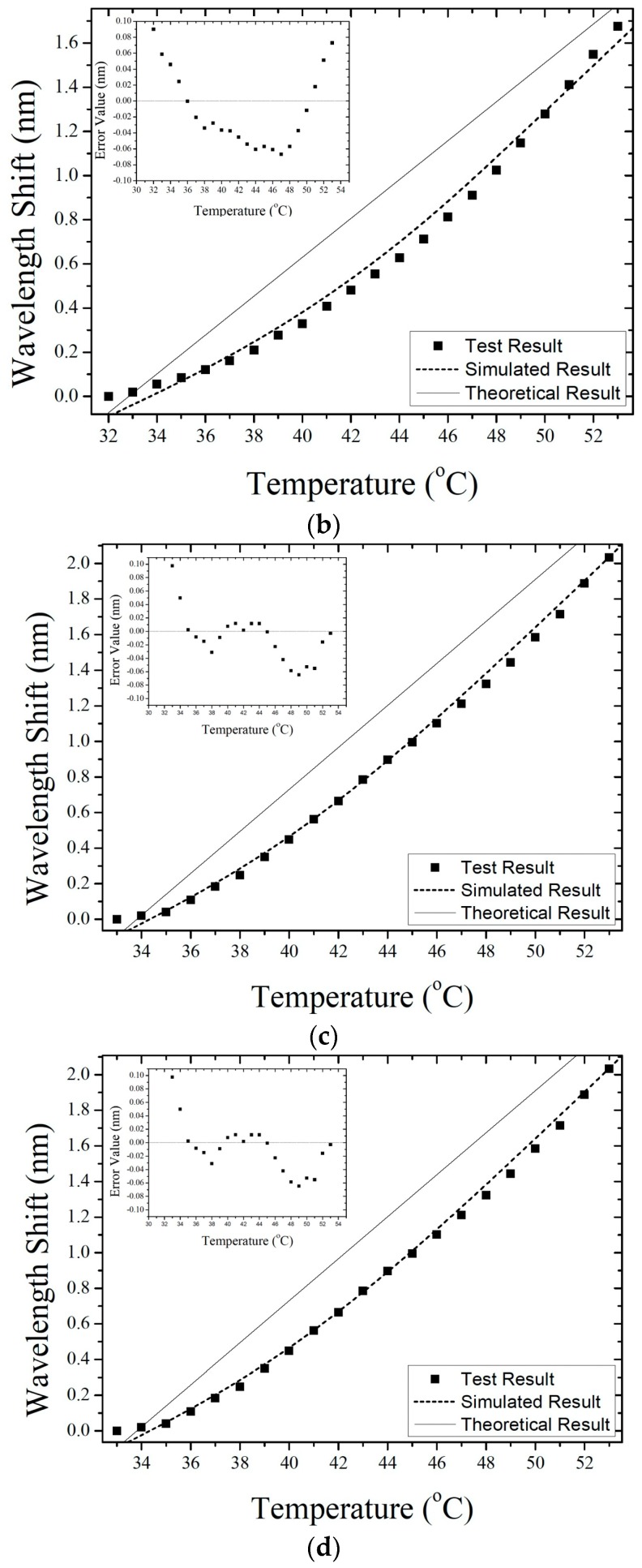
Wavelength shift versus temperature for different intracavity pressures: (**a**) 0.01 MPa; (**b**) 0.03 MPa; (**c**) 0.04 MPa; (**d**) 0.05 MPa.

Sensors with different gas pressures inside the FP cavity were subjected to temperatures from 32 to 53 °C in increments of 1 °C. This low temperature range was mainly based on the experimental object that is the body temperature of animals. Figure 10 shows an optical-microscope photograph of the fabricated FP sensor, and Figure 11 shows the reflectance spectra of the sensor with an intracavity pressure of 0.03 MPa at different temperatures. Figure 12a,d compare the experimental results with the simulation results for wavelength shift versus temperature at cavity pressures of 0.01, 0.03, 0.04, and 0.05 MPa. Overall, the simulation results are closer to the experimental results than the results calculated from theory. The small diagrams in the upper left corners of these four figures show the difference between the simulation results and experimental results, which indicate that when the pressure in the cavity is 0.01 MPa, the wavelength shifts of the simulation are less than the experimental shifts, with the difference increasing as the temperature increases. This result may be related to the greater effect of thermal stress on the elastic deformation of the silicon diaphragm at low intracavity pressure. Figure 12b,d show that, at higher pressures, the simulated values are consistent with the experimental values. The simulated results are less than the experimental results in some temperature ranges and are greater in other temperature ranges. For the intracavity pressure of 0.05 MPa, the curves are nearly straight lines. The temperature sensitivities of the four sensors with intracavity pressures of 0.01, 0.03, 0.04, and 0.05 MPa are 0.037, 0.080, 0.104, and 0.116 nm/°C, respectively. 

## 6. Conclusions

The results of this work elucidate the properties of silicon-diaphragm-based fiber-optic FP temperature sensors with various intracavity pressures. Simulations of the thermal stress and thermal expansion of the silicon diaphragm due to anodic bonding were done by using the heat transfer simulation and the solid mechanics simulation of finite-element analysis. The thermal stress is largest in the center and near the edges of the resonance cavity, reaching from 2 to 6 MPa. In addition, the reflection spectrum and the wavelength shift versus temperature of the sensor were simulated by introducing the thermal expansion results at different temperatures into the wave-optics module. The simulated temperature sensitivities of FP sensors with intracavity pressures of 0.01, 0.03, 0.04, and 0.05 MPa were 0.032, 0.087, 0.107, and 0.125 nm/°C, respectively. We also modeled the FP cavity by theoretical calculations that do not consider deformation of the silicon-diaphragm or thermal stress. Four FP sensors were subjected to temperature-variation experiments, and the results were compared with those of the finite-element analysis. In the case of high intracavity pressure of the FP sensor, the simulation results are consistent with experiment.
